# Remote, Disconnected, or Detached? Examining the Effects of Psychological Disconnectedness and Cynicism on Employee Performance, Wellbeing, and Work–Family Interface

**DOI:** 10.3390/ijerph20136318

**Published:** 2023-07-07

**Authors:** Laura Petitta, Valerio Ghezzi

**Affiliations:** Department of Psychology, Sapienza University of Rome, Via dei Marsi 78, 00185 Rome, Italy; valerio.ghezzi@uniroma1.it

**Keywords:** workplace disconnectedness, cynicism, performance, wellbeing, work–family interface, in-person and flexible workers

## Abstract

Owing to the COVID-19 pandemic, organizations worldwide have implemented remote working arrangements that have blurred the work–family boundaries and brought to the forefront employees’ sense of disconnectedness from their workplace (i.e., organizational disconnectedness) as a concern for multiple organizational outcomes. Cynicism, a job burnout subdimension, refers to a negative and excessively detached response to relational overload at work. While both workplace disconnectedness and cynicism involve a toxic sense of detachment, they refer to different psychological mechanisms. The present study aims to examine how employee workplace disconnectedness differs from their cynicism, and how both constructs differentially exert their detrimental effects on employee performance, work–family interface, and wellbeing. Using anonymous survey data collected online in 2021 and 2022 from a sample of in-person and flexible workers nested within organizations, conceptual distinctiveness between workplace disconnectedness and cynicism was supported. Measurement invariance across the two groups was supported, and subsequent structural invariance analyses suggested a similar pattern of results for flexible and in-person workers. Specifically, compared to disconnectedness, cynicism exerted higher negative effects on mental health and higher positive effects on cognitive failures and family-to-work conflict. Conversely, compared to cynicism, disconnectedness exerted higher negative effects on performance and work-to-family conflict. That is, feeling indifferent toward others particularly affects mental health and errors, while feeling excluded especially hampers productivity and family life. Theoretical and practical (e.g., inclusive leadership, support groups) implications of these results are discussed in light of the globally rising rates of hybrid work arrangements and related costs for employee wellbeing and productivity.

## 1. Introduction

According to the World Health Organization [1], disconnectedness and social isolation are among the contextual factors that contributed to the development of people’s stress and mental health issues during the COVID-19 pandemic. In Europe, four out of 10 workers (44%) say that their work stress has increased as a result of the pandemic [2], and remote working arrangements that cause being away from the workplace have only exacerbated employees’ feeling of loneliness and isolation [3]. *Workplace* or organizational *disconnectedness* (workplace disconnectedness and organizational disconnectedness are synonyms and are used interchangeably in the current paper) refers to the employee feeling different and distant from other people in the workplace, the frustration or disappointment by the failure of others to understand them, and the struggle to feel included in a valued group and connected with others [4]. Such feelings of distance and disconnectedness from the organizational community and lack of attachment to their work system and colleagues [5] represent the negative side of belongingness or, rather, the fundamental human need to be part of a group, the sense that one is part of something, and feeling attached to, close to, and thoroughly accepted by people in a community. On a different yet related note, *cynicism* is a component of job burnout and refers to an employee’s negative or excessively detached response to various demanding aspects of their job that cause an emotional and callous distance from one’s work [6]. Noteworthily, while both workplace disconnectedness and cynicism involve an employee’s feeling of detachment, the former involves the socially grounded and fundamental human *need* to bond with others, whereas the latter refers to a negative *attitude* toward one’s job and workplace developed in response to overwhelming job demands.

As the pandemic has shown, employees with a strong sense of belonging are over six times more likely to be engaged than those who do not [7,8,9]; thus, workplace disconnectedness has increasingly become an issue for organizations [10]. Similarly, workers surveyed by the American Psychological Association (APA) [11] reported heightened rates of burnout in 2021, which the World Health Organization (WHO) [12] suggested to increase mental distance from one’s job or feelings of negativism or cynicism related to one’s job.

Despite the widespread interest in employee workplace disconnectedness, empirical studies on its consequences for employee health and productivity, as well as work–family interface, are still scarce. Moreover, to date, no study has addressed the conceptual distinctiveness between workplace disconnectedness and work-related cynicism. Our goal in filling this gap is threefold. First, we seek to examine the conceptual distinction between employee workplace disconnectedness and work cynicism. Second, the present study aims at investigating the extent to which disconnectedness and cynicism differentially exert their detrimental effects on employee performance, work–family interface, and well-being. Third, our research aims at assessing the role of working arrangements (i.e., in-person vs. flexible) as a moderator of the hypothesized nomological network.

**Figure 1:**
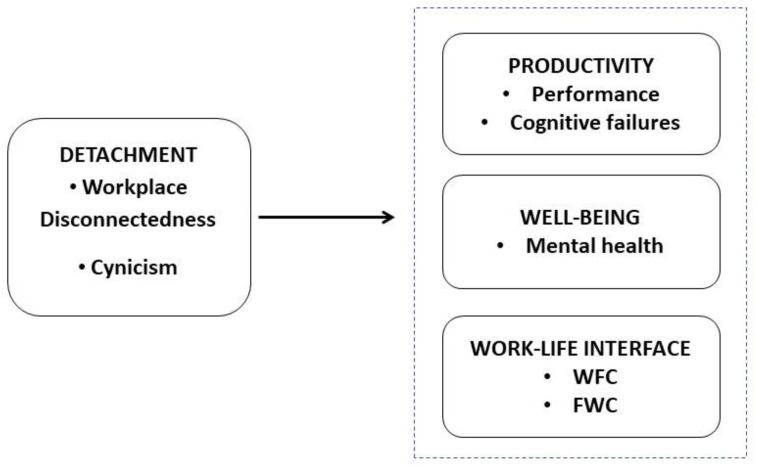
Conceptual model.

Using the belongingness literature [4] and job burnout literature [13] as a theoretical framework, we test the proposition that: a) higher workplace disconnectedness (i.e., the feeling of exclusion from the organizational system and lack of attachment to coworkers) [14] and accompanying feelings of isolation may lead employees to withdraw from common community purposes and tax their functioning, and b) higher work cynicism (i.e., a psychological detachment and a negative attitude about one’s work) [15] may drain employees’ emotional energy. Therefore, both workplace disconnectedness and cynicism are associated with reduced in-role behaviors (i.e., performance) [16], more hampering interferences when carrying out the work task (i.e., cognitive failures) [17], more difficulties in juggling role obligations in the work and family domains (i.e., work-to-family conflict and family-to-work conflict) [18], and lower employee mental wellbeing (i.e., mental health) [19]. Lastly, we examine whether in-person vs. flexible working arrangements may moderate the nomological network linking workplace disconnectedness and cynicism with employee productivity, wellbeing, and work–family interface. On the one hand, research suggests that flexible work arrangements may be associated with employees’ improved productivity and work–family interface. On the other hand, hybrid working may also make employees feel isolated and excluded from the social coworker network as compared to employees who work in-person, who are likely to have more occasions for spontaneous relationships, that may prevent the person to distancing themselves from others and the drift toward a solitary life [20,21,22]. Figure 1 shows the proposed nomological network linking the study variables.

In testing these propositions, our study provides some contributions to the literature. From a theoretical point of view, the present study integrates the belongingness literature (e.g., [4,14]) with job burnout theory [13] and is the first to examine the conceptual and empirical distinctiveness of workplace disconnectedness and work cynicism as two different yet related forms of unwanted employee detachment. Moreover, we add to the literature by assessing the role of workplace disconnectedness in the impairment of employee productivity and wellbeing, as well as the balance between work and family domains. Furthermore, the present study seeks to understand the role of working arrangements (i.e., flexible vs. in-person) in moderating the conjoint effects of disconnectedness and cynicism on health- and productivity-related outcomes within organizations. As such, we contribute to bridging the literature in the disparate field of well-established occupational health and the emerging field of belongingness at work. From a practical standpoint, our findings may assist scholars and practitioners to better understand the nuances of toxic employee detachment that may take the form of employees’ sense of exclusion and emotional distance from their organizational system (i.e., workplace disconnectedness), leading to self-isolation or a negative and disinterested reaction toward one’s work so as to avoid excessively demanding social interactions that mat translate into a self-serving distancing defense. Relatedly, our study provided suggestions on how to develop employee psychosocial resources such as the fulfilling sense of social and emotional connectedness with their organizational community.

Below, we begin our review of the literature by addressing the concept of workplace disconnectedness and work cynicism. Next, we review the literature on workplace disconnectedness and cynicism and address their conceptual similarities and differences. Moreover, we provide a theoretical background on the relationship of workplace disconnectedness and cynicism with employee productivity, wellbeing, and work–family interface. Lastly, we provide arguments regarding whether working in-person vs. flexibly may affect the proposed overarching nomological network among the study variables.

## 2. Employee Detachment and Its Relevance for Organizational Settings

While employee psychological detachment from work *when not at work* may be considered a resourceful process of ‘switching off’ when outside working time in order to mentally and physically distance oneself from work to enable recovery [23,24], employee detachment *when at work* may be considered a disinterested approach to the life of the organization, thus representing a toxic phenomenon of lack of involvement and distancing from their organization and work when dedication should be inherent. The present paper focuses on workplace disconnectedness [4,5] and work cynicism [6] as two different forms of employee detachment. Below, we first introduce the two constructs and then address their conceptual differences.

*Workplace disconnectedness* (or *organizational disconnectedness*) refers to an employee’s feeling of lack of unity and connectedness with their organizational community and lack of attachment to their work system and colleagues [5,25]. According to the literature [21], the sense of connectedness emerges during childhood and extends throughout adult life. It reflects the fundamental human need to establish significant relationships, be accepted by others in valued groups, and feel comfortable and confident within a larger social context. Such a sense of connectedness allows people to maintain feelings of being “human among humans” and refers to the need for (and sense of) belongingness. The need to connect drives people to seek social networking and helps identify those who may be different from themselves but share some point of commonality, which translates into feelings of being part of something more important than oneself (i.e., a community). Consistently, people strive to belong to a valued group and desire to be accepted by others [26]. Such a feeling of being part of a greater whole gives to the person a sense of purpose and brings satisfaction and security [27]. Moreover, the sense of belongingness distinctly applies to different domains of human life (e.g., family, school, and work) because every social setting has unique and nonoverlapping attributes. In other words, the sense of connectedness is context-specific and uniquely captures the essence of that social setting such as the domain of organizational settings [25]. Noteworthily, social disconnectedness (e.g., workplace disconnectedness) appears when the person begins to feel different and distant from other people and struggles to feel connected [4], thus failing to fulfill the natural desire to belong, thereby experiencing isolation. When a person tries to relate with others but gets frustrated or disappointed by the failure of others to understand him or her, then s/he may find it hard to accept social roles and responsibilities, thus distancing themself from the community and developing a sense of disconnectedness.

Job burnout is a form of work-related stress and refers to an employee’s response to overwhelming demands that develops progressively and can eventually become chronic [6]. While, during the initial stage of the burnout process, employees feel that their emotional resources are depleted by overwhelming work and contextual demands, their subsequent attempts to cope with such emotional exhaustion lead them to defensively withdraw from work and fall into negative and cynical reactions. Work *cynicism* refers to a state of psychological detachment and a negative attitude about one’s work and workplace [15]. Workers who self-protect from the overload of interpersonal strain also tend to progressively develop a negative reaction to people and to the job over time, and drift toward detachment from their work and the workplace. The cynicism component of burnout represents the interpersonal context dimension of burnout and places the individual strain experience within the social, relational context of the workplace [28]. Specifically, it refers to a negative, callous, or excessively detached response to various demanding aspects of one’s job and to the process of distancing oneself emotionally and cognitively from one’s work. According to Leiter and Maslach [29], being burned out from one’s job does not simply imply being exhausted or overwhelmed by workload but also includes having lost a psychological connection with one’s work. Specifically, cynicism captures employees’ disaffection with work.

Overall, workplace disconnectedness and cynicism are constructs sharing some commonalities. First, they are both phenomena that involve an employee’s *distancing reaction* in response to unwanted work experiences. Thus, they similarly refer to a form of employee detachment. Second, a *social*/*relational component* is inherently embedded in both workplace disconnectedness (i.e., the feeling of isolation due to lack of acceptance from others) and cynicism (i.e., the callous distancing from demanding interactions at work).

Despite some similarities, workplace disconnectedness and cynicism are conceptually distinct. First, while workplace disconnectedness refers to the frustration of a basic human *need* (i.e., the desire to affiliate and belong), work cynicism is a negative *attitude* toward work developed as a defensive response to the depletion of emotional resources. Second, workplace disconnectedness is a *self-isolating* social response to the lack of inclusion from others and involves the relational dimension in terms of attachment, whereas cynicism is a depersonalizing and negative feeling toward others when their emotional demands are too demanding for the employee; thus, it is a *self-serving* social response. Third, while both constructs involve a social component of interactions with others at work, disconnectedness is a form of detachment from *a valued community* and the organization as a whole, whereas cynicism is the distancing experienced *toward one’s work* (and demanding people), which becomes less valuable in the eye of the burned-out employee. Below, we address in detail the role of workplace disconnectedness and cynicism as different forms of employee detachment in predicting employee productivity and wellbeing, as well as work–family interface.

### 2.1. Workplace Disconnectedness and Cynicism as Predictors of Employee Work–Family Interface, Productivity, and Wellbeing

Workplace disconnectedness refers to an employee’s feeling of distance from the organizational community and a lack of attachment to their work system and colleagues [5]. Such a sense of disconnectedness stems from the frustration of the desire to belong or the fundamental human need to be part of a group, the sense that one is part of something, and feeling attached to, close to, and thoroughly accepted by people in a community [4]. According to the literature, the need to belong is a basic emotional need to affiliate with others, thus boosting the individual’s intrinsic motivation to be part of something more important than themself [30]. Such a human desire for connection drives people to seek out for companionship and participate to a common purpose of a community [31]. Indeed, research in organizational settings [32] suggests that employee belongingness is associated with a 56% increase in job performance, thus demonstrating that the sense of belonging is a key ingredient for performance at work. Not only is the desire to be accepted by the organizational community a greater motivator than money that drives employees to engage more effectively and perform better [33], but a sense of social belonging can also affect intellectual achievement (e.g., cognitive failures).

Cognitive failures at work are breakdowns in cognitive processing of one’s job task that a person should normally do without mistakes, as well as unintended execution lapses stemming from problems with attention (i.e., failures in perception), memory (i.e., failures related to information retrieval), or action (i.e., the performance of unintended actions or action slips) [17,34]. Such cognitive failures are departures from functional cognitive operation, constituting cognitively based errors. Indeed, the literature [35] suggests that people’s sense of belonging and reliance on group members affect their cognitive abilities and mental states, which boost their coping with tasks and challenging situations. While the feeling of being accepted by a group may increase the development of skills for understanding the mental states of their social partners (e.g., coworkers), an employee’s sense of exclusion from a community may increase feelings of loneliness and isolation, as well as boost negative emotional states of insecurity, lack of acceptance, unpleasant social interactions, and lack of engagement [4,30]. Thus, while positive emotional states (e.g., belongingness) enhance cognitive functioning through a perceived ability to control the course of events, positive thinking, and enhanced capacity of discernment [36,37,38], negative emotional states (e.g., sadness that ensues from isolation and workplace disconnectedness) may impair overall cognitive functioning and information processing, which prevent the risk of engaging in off-task behaviors (i.e., cognitive failures) [39].

As noted, workplace disconnectedness refers to the employee feeling different and distant from other people in the workplace, thus representing a sense of frustration with or disappointment in the failure of others to understand them, which causes a struggle to feel included in a valued group and connected with others [4,5]. While there is currently no study on the effects of workplace disconnectedness on employee performance and cognitive failures at work, on the basis of the above arguments, we may speculate that employees’ feeling of lack of acceptance by their coworkers boosts negative affective and emotional states associated with a lack of inclusion and attachment (i.e., disconnectedness), thus hampering their motivation to participate in a common purpose within a community [31] and worsening their ongoing cognitive processing of on-task activities and intellectual achievement (i.e., cognitive failures).

Extensive research and metanalytic findings suggest that job burnout depletes employees’ energies and determines poor performance (e.g., [40,41,42]). Specifically, workers experiencing burnout are not simply tired; they are discouraged and alienated [43,44], and cynicism is the more distinctive and central aspect of burnout because this is where the work experience goes wrong. Indeed, cynicism stems from a poor quality of social relationships at work and a lack of critical resources, all of which can lead to poor job performance [29]. Relatedly, while the literature on job burnout and cognitive failures is still scarce, research suggests that burned out individuals often complain about attentional problems, and burnout was found to be associated with difficulties in voluntary control over attentional tasks; furthermore, the level of such cognitive deficits varied with the severity of burnout symptoms (e.g., [45,46]).

Thus, if the above arguments are supported, we would hypothesize the following:

**Hypothesis** **1a:** *Workplace disconnectedness negatively predicts job performance.*

**Hypothesis** **1b:** *Workplace disconnectedness positively predicts cognitive failures.*

**Hypothesis** **2a:** *Cynicism negatively predicts job performance.*

**Hypothesis** **2b:** *Cynicism positively predicts cognitive failures.*

Moving to the link of workplace disconnectedness with employee wellbeing, the literature [14,31] suggests that people’s happiness is inextricably tied to the need for other people and their acceptance, and that people’s sense of belonging is inextricably tied to their physical and mental health. Indeed, anxiety (i.e., the experience of a set of affective and behavioral states characterized by tension, apprehension, and agitation) and depression (i.e., a deflection of mood and a pessimistic and discouraged orientation toward existence) are two major components of an individual’s mental health [19]. Noteworthily, depression and anxiety are both common health impairment conditions associated with loneliness and the lack of a sense of belonging to a group (i.e., disconnectedness), as well as sharing of common interests and aspirations [31,47]; research in work settings [48,49] supports the role of belongingness as a predictor of employee depression and anxiety disorders (i.e., mental health). Moreover, the neuroscience literature suggests that people crave interactions in the same region of the brain where they crave food, and their happiness and wellbeing are inextricably tied to the need for acceptance and the feeling that they belong to a greater community [50]. While belonging to a group and working with other people may enhance feelings of inclusion and yield emotional support in the face of difficulties (e.g., coworkers can console one another), feelings of disconnectedness and isolation may weaken the individual when coping with hardships and related negative physical and mental effects of such challenging situations [51,52].

Burnout syndrome is an individual response to chronic work stress that develops progressively and includes a picture or set of symptoms and signs that cause damage at a cognitive, emotional, and attitudinal level. Cynicism, the interpersonal component of burnout, includes feelings of detachment and indifference toward one’s work and/or the people who receive it; it is, thus, associated with irritability and interpersonal avoidance, which inevitably wear out an employee’s psychological resources [53]. As such, numerous studies have demonstrated how the syndrome translates into negative behavior toward work, peers, users, and the professional role, ultimately taxing the employee’s mental energy and causing health problems [54]. In particular, research findings (e.g., [29,40]) suggest that psychological alterations generated by the syndrome of being burned out at work and the accompanying reduced coping capacities are associated with symptoms of increased physiological activation and anxiety caused by stressful stimuli, as well as alarming levels of depressive feelings of lacking the emotional energy to face the working day (i.e., poor mental health) [19].

On the basis of the above arguments, we pose the following hypotheses:

**Hypothesis** **3a:** *Workplace disconnectedness positively predicts mental health.*

**Hypothesis** **3b:** *Cynicism positively predicts mental health.*

The work–family interface refers to how the work and family life domains reciprocally influence each other, and whether employees can simultaneously engage in both work and family responsibilities or, conversely, experience conflicting demands associated with their different roles as workers and family members [18]. Role stress theory suggests that such conflict between the two interrelated roles may occur when pressures on the roles within the work and family domains are incompatible and/or contradict each other, and when concurring responsibilities are difficult to reconcile, thereby creating more demands than the employee can handle [55,56]. Specifically, work-to-family conflict (WFC) is defined as the conflict that the person experiences when the different roles within their work life and family life impose conflicting expectations, and when work commitments tend to interfere with family life and obligations. Similarly, family-to-work conflict (FWC) is the conflict experienced by workers when the boundaries between work and private life become blurred, and when family responsibilities jeopardize their engagement in working activities.

As noted above, workplace disconnectedness is the feeling stemming from the lack of acceptance and inclusion from others at work and lack of emotional significance attached to a valued group membership, as well as the frustration related to the desire to form and maintain social bonds. While, to date, there are no studies on (lack of) belongingness and WFC or FWC, the literature suggests that belonging is the first core social motive [57], and that belonging to a group stimulates prosocial behavior toward the in-group, thus serving as a psychological resource that helps coping with hardships that stem from hampering situations [26,31] and building resilience [58]. As such, lack of emotional attachment to a workplace community and the related shortage of a sense of membership arguably deprive workers of psychological resources that may help in building resilience and dealing with the stressful difficulties of juggling conflicting work and family demands (i.e., WFC and FWC).

Thus, we hypothesize the following:

**Hypothesis** **4:** *Workplace disconnectedness positively predicts (4a) WFC and (4b) FWC.*

Cynicism is a dimension of burnout and represents the opposite of a sense of an employee’s dedication to and involvement in their work; it is characterized by feelings of lack of enthusiasm and significance [59]. Abundant studies suggest that increased job requirements may limit the ability of employees to effectively participate in their work and family roles, and such role conflicts can lead to work interfering with family life, and vice versa (i.e., WFC, FWC), which depletes employees’ energy and is associated with an increased risk of burnout [29,40]. While work–family conflict may increase employees’ work stress and trigger job burnout, research also suggests a recursive effect of work-related stress on demanding situations such as the work–family interface in less engaged employees (e.g., less dedication and cynical attitude), who are also less able to handle job demands such as the blurred boundaries between work and family domains [60].

On the basis of the above arguments, we pose the following hypothesis:

**Hypothesis** **5:** *Cynicism positively predicts (5a) WFC and (5b) FWC.*

### 2.2. Do Working Arrangements Matter for Employee Detachment and Its Outcomes?

Due to the COVID-19 pandemic, organizations around the globe have implemented remote working during lockdowns and have continued to use flexible work models including hybrid solutions incorporating both on-site and home-based arrangements. As noted, the OSHA [3] has reported higher workplace stress due to loneliness and feelings of isolation at work, most likely resulting from being away from the workplace and distancing from colleagues. In addition, the EU-OSHA [2] noted that poor work organization and a negative social context at work are associated with increased psychosocial risks for employee health. Similarly, research has suggested that, while the advantages of hybrid work schemes include better work–life balance and productivity, disadvantages involve increased feelings of isolation [22]. Working alone can make some workers feel cut off from their colleagues, thus highlighting the relevance of implementing hybrid models that best suit employees and help counteract the sense of disconnectedness [61]. As such, alternative work arrangements may lead many workers to feel they do not formally belong to the organization (i.e., workplace disconnectedness), as well as make it harder for on-site employees to feel a sense of unity with colleagues who work from home. Moreover, the use of technology-mediated communication and remote interactions leads to scheduled interactions and prevents employees from taking part in spontaneous social contacts, as well as developing meaningful relationships that may help build a sense of belongingness and inhibit feelings of alienation [20,21]. Additionally, the Chartered Institute of Personnel and Development (CIPD) [62] indicates that nearly half (48%) of organizations surveyed in the United Kingdom report being concerned about potential feelings of a lack of belongingness that may arise from a move to hybrid or home working, while one-quarter (24%) of employees are concerned about being treated less favorably in comparison to colleagues who work on-site.

Within the existing literature, social connection has been deemed essential to an individual’s feelings of belonging to a valued group, and physical proximity allows for the creation and maintenance of social bonding [63]. Undoubtedly, working on-site consents physical closeness and the opportunity for frequent and spontaneous interactions that facilitate not only interpersonal relationships [64] but also emotional attachments to individuals within one’s work context [65]. In terms of the effects of working arrangements on burned out employees, cynicism represents the social component of the syndrome that stems from emotionally demanding interactions with other people at work (e.g., service users) that wear out an employee’s psychological energy and translate into detached and indifferent attitudes and behaviors toward others [15]. On the one hand, one may argue that remote working arrangements likely boost employees’ sense of distance from others at work, thus potentially contributing to enhanced cynicism and related outcomes. Indeed, research suggests that the impact of the pandemic on remote work burnout has been staggering, and employees feel as if they have no emotional support from their employers [66]. On the other hand, it may be speculated that working remotely and being away from the demanding relationships at work that originally caused the detachment reaction of employees likely removes the problem and, thus, may contribute to attenuate employees’ cynical symptoms.

On the basis of these arguments, we might arguably expect a differential pattern of relationships among our study variables (i.e., workplace disconnectedness, cynicism, productivity, wellbeing, and work–family interface) in the “in-person” vs. flexible (i.e., remote and hybrid) group of workers. To date, we could not find published studies examining the moderating effect of working arrangements on the hypothesized nomological network. While the existence of previous significant knowledge allows making specific predictions and developing a specific hypothesis on the link between two variables, the lack of significant contributions does not allow making any specific claim, and only allows more cautiously developing an explorative research inquiry. This is particularly relevant when simultaneously testing multiple links of an overarching nomological network among numerous variables, as is the case in our study. Thus, we pose the following research question:

*Research Question:* Do working arrangements (in-person vs. hybrid) moderate the relationship of both workplace disconnectedness and cynicism with employee productivity (i.e., in-role performance and cognitive failures) and wellbeing (i.e., mental health), as well as work–family interface (i.e., WFC and FWC)?

## 3. Method

### 3.1. Participants and Procedure

The sample consisted of 1066 workers from 204 organizations in Italy. Data on the organizations’ size were not collected; therefore, an estimated total population for the 204 organizations is not available. Participants were equally divided between males (50.2%) and females (49.8%). The average age of respondents was 41.9 years (*SD* = 13.20), with a range of 18–67. The slight majority (53.3%) held a fixed-term position within their organization. The average tenure in the position was 11.4 years (*SD* = 10.41), with a range from <1 year to 40 years. Education of respondents was mainly at the college (41.6%) and high-school (27.1%) levels. Organizations were recruited from the following industry sectors: healthcare (12%), education (19.8%), hotel (11.1%), manufacturing (1.3%), commerce (6.1%), transportation (3.4%), communication and technology (10.6%), military (2.8), construction (2.1%), services and finance (5.3%), and unspecified (25.5%).

We collected online anonymous survey data via Survey Monkey from a convenience sample of Italian adult workers. The research team reached potential participants belonging to the same organization to request their participation in the study, provide information to describe the project, encourage participation, and address concerns. Data were collected in 2021 and 2022. While COVID-19 containment measures were in place during this period, the questionnaire was accessible online, and participants were contacted in a manner compatible with all sanitary restrictions. Participation was voluntary, anonymous, and not rewarded by any incentive. The study followed the guidelines of research ethics. Participants were provided with informed consent materials that explained the anonymous nature of the data collection and their rights as research participants. Moreover, the survey included five quality check items to detect careless responding.

### 3.2. Measures

Below is a description of measures used to provide data for the current analyses. Specifically, for the cognitive failure [39] and cynicism [67] scales, we used the previously translated Italian versions. The workplace disconnectedness [4], in-role performance [16], mental health [19], work-to-family conflict, and family-to-work conflict [68] scales were translated into Italian from the English version using the standard translation/back-translation procedure recommended by Brislin [69]. The correspondence of the original and the back-translated items was then verified by the authors.

#### 3.2.1. Workplace Disconnectedness

Workplace disconnectedness was assessed using eight items of the social connectedness scale [4], adapted to organizational contexts. The adapted items are intended to capture the feelings of employee psychological detachment from their own organization and colleagues. A sample item is the following: “I feel disconnected from the organization I work for”. Respondents were asked to rate their degree of agreement with the proposed statements, using a five-step Likert scale (1 = strongly disagree; 5 = strongly agree).

#### 3.2.2. Cynicism

We used the Italian version [67] of the Maslach Burnout Inventory—General Survey (MBI—GS) [70] to assess cynicism. Five items measured employee feelings of callous detachment from one’s work. A sample exhaustion item is the following: “*I am becoming more detached from my work*”. Items were rated on a seven-point frequency scale ranging from *never* (0) to *daily* (6).

#### 3.2.3. Performance

Three items measured employee performance of in-role behaviors as behaviors that are formally recognized and are part of the job requirements as expected by the job description [16]. A sample item is the following: “*I fulfill the responsibilities specified in my job role*”. Items were responded to on a five-point Likert-type frequency scale (1 = never; 5 = always).

#### 3.2.4. Cognitive Failures

We used the Italian version [39] of the workplace cognitive failure scale, developed by Wallace and Chen [17], to measure cognitively based errors that occur during the performance of a task. Nine items measured three components of workplace cognitive failures: (a) memory (i.e., information retrieval failures; sample item: *You forget where you have put somethings that you use for work (e.g., instruments)*)*;* (b) attention (i.e., failures in perception; sample item: *You do not fully concentrate your attention on working activities*); (c) action (i.e., performance of unintended actions; sample item: *You say things to others which you did not intend to say*). Respondents were asked to indicate their agreement using a five-point Likert scale ranging from 1 (strongly disagree) to 5 (strongly agree). Items were averaged to reflect an overall measure of cognitive failures at work.

#### 3.2.5. Mental Health

Three items from the Veit and Ware’s scale [19] assessed respondents’ psychological distress. A sample item is the following: “*During the past month, how often have you been a very nervous person?*”. All items were responded to on a five-point Likert-type frequency scale (1 = none of the time; 5 = all of the time). Negative items were reverse-coded such that higher scores reflected greater levels of psychological wellbeing.

#### 3.2.6. Work–Family Interface

We used the short version of the scale from Matthews, Kath, and Barnes-Farrell [68] assessing (a) the extent to which work commitments interfere with family life (i.e., WFC; sample item: “*I am often so emotionally drained when I get home from work that it prevents me from contributing to my family*”, and (b) the extent to which family obligations interfere with work life (i.e., FWC; sample item: “*I have to miss work activities due to the amount of time I must spend on family responsibilities*”). All items were responded to on a five-point Likert-type scale (1 = strongly disagree; 5 = strongly agree).

### 3.3. Analytical Strategy

In order to maximize the reliability and parsimony of our structural equation model, item parcels were created for construct measures with more than three items (i.e., disconnectedness, cynicism, mental health, and cognitive failures). We followed Little, Cunningham, Shahar, and Widaman’s [71] and Little, Rioux, Odejimi, and Stickley’s [72] recommendations and sequentially assigned items on the basis of the highest to lowest corrected item-to-scale correlations to create three item parcels per construct. Subsequent analyses were conducted using the parcels as manifest indicators of the latent variables with *Mplus* 8.0 [73]. Moreover, in the current study, people were nested within organizations; therefore, the resulting data were hierarchical in nature and non-independent. Because such data may result in artificially low estimates of standard errors, we used the “type = complex” procedure of MPLUS to rectify this [73]. This approach produces correct parameters estimates, standard errors, and test statistics in the presence of clustering of individual observations [74].

## 4. Results

### 4.1. Descriptive Statistics

Means, standard deviations, alpha coefficients, and zero-order correlations among the scales are reported in Table 1. As shown in the diagonal of this table, each study variable had a good degree of internal consistency reliability (composite and maximal reliabilities), with the exception of WFC that displayed slightly low values (a composite reliability of 0.61 and a maximal reliability of 0.63), suggesting the importance of controlling for measurement error in future models for this construct [75].

#### 4.1.1. Goodness of Fit for the Measurement Models of the Single Groups

Prior to conducting multiple-group analyses for testing our hypotheses, we examined the goodness-of-fit values of the seven-factor CFA models separately for the *in-person* and *flexible* samples of workers. The values for the in-person sample (see Table 2) were as follows: Satorra–Bentler χ^2^_(df = 168)_ = 585.206, RMSEA = 0.058 (0.053−0.063), CFI = 0.93, TLI = 0.92, and SRMR = 0.046, showing a good fit. Similarly, the fit indices for the flexible sample were as follows: Satorra–Bentler χ^2^_(df = 168)_ = 323.936, RMSEA = 0.061 (0.051−0.071), CFI = 0.92, TLI = 0.89, and SRMR = 0.061, indicating an acceptable fit. These results demonstrated the appropriateness of the seven hypothesized latent factors and the distinctiveness of workplace disconnectedness and cynicism, as well as mental health, productivity factors (i.e., performance and cognitive failures) and work–family interface (i.e., WFC and FWC).

#### 4.1.2. Multiple Group CFA Analyses for Invariance across In-Person and Flexible Workers

Table 2 shows the results of analyses for measurement invariance testing. When constraints on factor loadings were added to test for metric invariance, the model (M2) still showed a good fit, and the ΔCFI was <0.01, in contrast to the configural model (M1). Overall, there was good evidence for the equality of loadings across the in-person and the hybrid samples of workers, and the metric level of invariance was appropriate for the purpose of the current study (comparing structural effects across subsamples), as it was not aimed at comparing the means of latent (or observed) variables [76].

### 4.2. Test of the Structural Model

**Figure 2:**
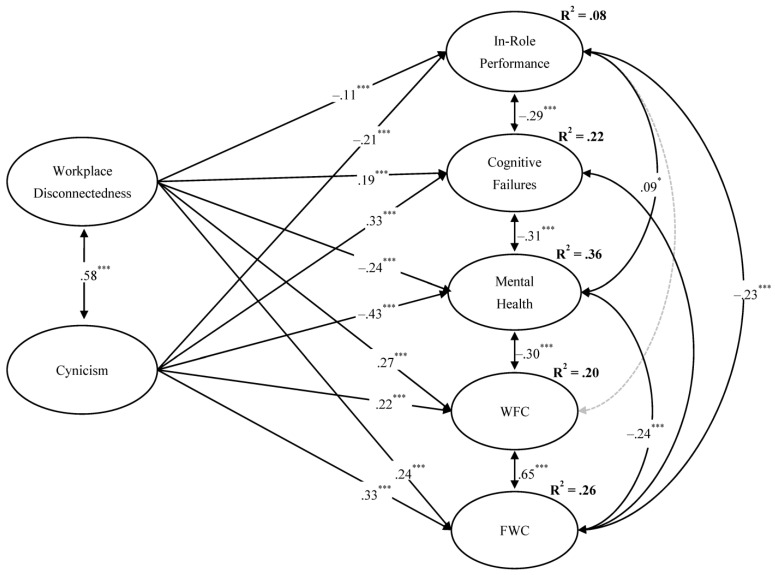
Standardized estimates for the final structural model. Note: Results are presented in a completely standardized metric; R^2^ values were all significant for all latent variables at *p* < 0.001 (*** *p* < 0.001; * *p* < 0.05).

In the first step, we examined the goodness-of-fit values for the structural equation models separately for the in-person and flexible workers. Results (see Table 2) from the comparison of single analysis across both in-person and flexible groups without any constraints (model S4) and with constrains imposed on structural effects (S5) showed that there was no significant decrement in model fit. As such, results demonstrated an invariant pattern of relationships among the latent variables across the in-person and flexible workers. The final best-fitting model of the total sample (Satorra–Bentler χ^2^_(df = 168)_ = 758.074, RMSEA = 0.058 (0.054−0.062), CFI = 0.93, TLI = 0.91, and SRMR = 0.046) is presented in Figure 2. As can be seen, workplace disconnectedness and cynicism both negatively predicted in-role performance (−0.21, *p* < 0.001 and −0.11, *p* < 0.001, respectively), thus lending support to Hypotheses 1a and 2a; disconnectedness exerted a higher effect in comparison to cynicism. Additionally, disconnectedness and cynicism both positively predicted cognitive failures (0.19, *p* < 0.001 and 0.33, *p* < 0.001, respectively), thus lending support to Hypotheses 1b and 2b; cynicism exerted a higher effect in comparison to disconnectedness. In general support of Hypotheses 3a and 3b, workplace disconnectedness and cynicism both negatively predicted mental health (−0.24, *p* < 0.001 and −0.43, *p* < 0.001, respectively); cynicism exerted a higher effect in comparison to disconnectedness. Lastly, disconnectedness and cynicism both positively predicted WFC (0.27, *p* < 0.001 and 0.22, *p* < 0.001, respectively), thus lending support to Hypotheses 4a and 5a; disconnectedness exerted a higher effect in comparison to cynicism. Interestingly, in support of Hypotheses 4b and 5b, disconnectedness and cynicism both positively predicted FWC (0.24, *p* < 0.001 and 0.33, *p* < 0.001, respectively); in this case, cynicism exerted a higher effect in comparison to disconnectedness. Table 3 shows a summary of the results of hypothesis testing.

Overall, cynicism and disconnectedness explained 8% of the variance in in-role performance, 22% of the variance in cognitive failures, 36% of the variance in mental health, 20% of the variance in WFC, and the 26% of the variance in FWC.

## 5. Discussion

Post-pandemic scenarios have witnessed an increase in work-related stress worldwide [2] and an increased use of remote working and flexible work arrangements (i.e., mixed in-person and remote working), which are not without risks to workers’ productivity and wellbeing [22,77]. Specifically, the employee’s sense of personal connectedness with the organization that makes them feel an integral part of the organizational system has been further challenged by remote working and being away from the workplace, which arguably foster feelings of isolation and loneliness that are potentially harmful for their health [3]. Despite the increased interest in organizational disconnectedness, empirical studies on its consequences for employee productivity and wellbeing are still scarce. The present study is the first to examine across groups of in-person and flexible workers how employee workplace disconnectedness differs from their cynicism, and how disconnectedness and cynicism differentially exert their detrimental effects on employee performance, wellbeing, and work–family interface.

Our findings from preliminary analyses on the measurement model suggest adequate psychometric properties of the workplace disconnectedness scale, as well as its distinctiveness from work cynicism, thus lending initial support to the conceptual distinction between workplace disconnectedness and cynicism as two different forms of employee sense of detachment. Next, results from the structural model analyses on multilevel and multigroup data supported all the hypothesized links among the study variables and suggested that both workplace disconnectedness and work cynicism predict poor employee productivity and wellbeing, as well as more conflict in the work–family interface. Specifically, compared to cynicism, workplace disconnectedness exerted higher negative effects on performance and positive effects on work-to-family conflict. Hence, when employees experience a lack of acceptance at work, this makes them feel less involved with the organizational system. As such, they fail to participate in the common meaningful purpose of the community and are less effectively engaged in performing their work tasks (i.e., performance), as well as lack the psychosocial resources in copying with the difficulties of juggling conflicting work and family demands. Similarly, cynicism and its negative attitude toward work negatively affect in-role performance, as proposed in the literature (e.g., [29,78]). Interestingly, lacking a sense of belongingness to the organization and the larger community seems to be an even more effective hampering factor on employee performance as compared to their indifference toward work and demanding others, thus representing a more critical facet of employees’ detachment regarding their performance. Relatedly, when employees sense a lack of belonging to a group and experience less relational involvement and support from others at work, they likely lack the psychosocial resources that may help in handling work obligations that interfere with private life. In this case, employees’ negative and cynical attitude toward work also has similar negative effects on the management of their work and family roles; however, a frustrated sense of belonging to a community (i.e., disconnectedness) seems to exert a more impactful effect on role conflict management as compared to their defensive response of detached interpersonal avoidance (i.e., cynicism). This is in line with the practitioners’ claim regarding the relevance of preventing employee detachment in the wake of the pandemic (e.g., [79]).

Our findings also suggest that, compared to workplace disconnectedness, work cynicism exerted higher negative effects on mental health and higher positive effects on cognitive failures and family-to-work conflict. This agrees with post-pandemic reports from the American Psychiatric Association [80], suggesting that people’s concerns regarding their mental health are on the rise, as demonstrated by nearly two out of five (37%) Americans rating their mental health as only fair or poor, up from 31% the year before, as well as one in four (26%) reporting that they anticipated experiencing more stress at the start of 2023, up from one in five (20%) the year before. Indeed, employees’ response of detachment and indifference due to unbearable emotionally exhausting relational demands at work is associated with feelings of emotional fatigue (e.g., [13]) that likely explain the higher impact of cynicism on employee depression and anxiety (i.e., mental health), more so than their sense of disconnectedness from the organizational community that comes along with lower feelings of happiness [14,31]. Relatedly, disinterest toward one’s work and accompanying irritation toward demanding others (i.e., cynicism) appear to impair employees’ executive control in cognitive processes, underlying the voluntary and effortful regulation of perception that underpins task activities (i.e., cognitive failures) [81], more so than their feelings of a lack of acceptance from others at work and isolation (i.e., disconnectedness). Lastly, the irritation and defensive distancing attitude of cynical employees associated with declined response inhibition may increase the likelihood of inappropriate behavior (e.g., not being able to withhold oneself during a conflict) [46], thus seeming to exert a more hampering effect on employees’ management of family demands interfering with work requirements in comparison to the feelings of a lack of inclusion from others at work (i.e., disconnectedness).

Moving to the role of working arrangements (i.e., in-person vs. flexible) as a moderator of the hypothesized relationships of both workplace disconnectedness and cynicism with employee productivity, wellbeing, and work–family interface, our findings suggest a similar pattern of relationship for employees working on site and flexibly. Overall, the more employees lack a sense of membership and attachment to their workplace, the more they are filled with feelings of isolation and loneliness. Additionally, the more employees are exposed to demanding relationships at work, the more they develop a distant and detached response. Both these conditions, whether employees work on-site or flexibly, worsen their productivity and increase their work-related distress in response to depleted emotional energy, as well as hamper their abilities to manage conflicting demands from work and family domains. As such, working in-person and remotely seems to equally provide occasions for toxic interactions and a lack of acceptance, which build employees’ detachment and related outcomes. On the one hand, social connection is essential to build a sense of belongingness to an organizational community [25], as well as develop social support that may attenuate employee cynicism [40], and our findings are in line with the literature suggesting that physical proximity is a key ingredient in creating and maintaining a social bond that builds people’s sense of belonging [63]. On the other hand, relational distance may also be experienced when employees are physically close, whereas geographically distant coworkers may feel emotionally close. In other words, social bonding may depend more upon “perceived” proximity (i.e., an individual’s perception of how close or how far another person is) rather than objective physical proximity [82].

### 5.1. Theoretical Implications

Our results have implications for the extant literature in the areas of occupational health, workplace disconnectedness (or lack of organizational belongingness), and job burnout. First, the results on the conceptual distinctiveness between workplace disconnectedness and work cynicism inform the occupational health literature by showing that employee psychological detachment *when at work* and resulting poor outcomes may take two different yet equally toxic forms. Specifically, while both constructs call into account an employee’s uncomfortable experience of psychological distance in response to workplace hardships related to social relations, workplace disconnectedness involves the frustration of a basic human *need* to belong to a greater whole [4] and translates into a *self-isolating* social response to other people’s lack of acceptance. Conversely, work cynicism encompasses a negative and distant *attitude* toward work that unfolds from defensive attempts to avoid excessively demanding social interactions at work [13] and translates into a *self-serving* social response. Noteworthily, both workplace disconnectedness and work cynicism predict poor employee productivity and wellbeing, as well as more conflict in the work–family interface. This seems to align with emerging trends on burnout studies suggesting that the current work arrangements increase the likelihood of emotional demands associated with adverse social behaviors [83]. Overall, organizations are warned against employee detachment that may take the form of employees’ feeling of disconnectedness *toward a valued community* and the organization, as well as the distance from *one’s work* (and demanding relationships), which becomes less valuable in the eye of a burned-out employee.

Second, our findings also inform the still nascent workplace disconnectedness (or organizational belongingness) literature by demonstrating that a lack of organizational membership is associated with lower employee productivity (i.e., lower performance and higher cognitive failures), poorer mental health, and increased role conflicts and difficulties in juggling the blurring of work–family boundaries (i.e., WFC and FWC). Specifically, the lack of companionship and acceptance from others at work underpins employees’ low investment in a common purpose of the wider organizational community, which translates into emotional dissatisfaction and demotivation to bring their best selves to work [5]. In turn, this eventually leads them to experience cognitive interferences and impaired functioning in performing their work task, as well as higher feelings of being emotionally drained by work pressures and perceived threats. Moreover, our findings showing how employees’ disconnectedness is associated with more conflicting demands across the work and life domains, which expands the role stress theory [18,55]. Specifically, we found evidence that employees’ frustration in their desire to form and maintain social bonds at work represents an additional taxing social and emotional demand that wears out their energy and makes them experience more work obligations interfering with their family life, and vice versa [56]. Relatedly, our findings also add to the job burnout literature by showing how employees’ cynicism and interpersonal detachment impair their cognitive functioning and increase their in-task errors (i.e., cognitive failures), thus providing additional evidence for a still understudied link (e.g., [84]).

Third, our study is the first to simultaneously examine the role of working arrangements (i.e., in-person vs. flexible) as a moderator of the hypothesized relationships of both workplace disconnectedness and cynicism with employee productivity, wellbeing, and work–family interface, thereby contributing to bridge the still disparate fields of occupational health, belongingness at work, and job burnout. Our findings suggest a similar pattern of relationship for employees working on site and flexibly, and show that employees experiencing distance from their workplace and lack of attachment to their coworkers (i.e., disconnectedness), as well as a callous indifferent attitude toward others (i.e., cynicism), are deprived of the necessary emotional energy to endure the efforts of effective productivity and to help flourish their wellbeing, while also weakening their coping with the juggling of mutually interfering work and family obligations. On the one hand, this is in line with the literature suggesting that social connection and bonding that build employees’ sense of belongingness to a valued group seem to depend more upon one’s perception of how close or how far another person is (i.e., perceived proximity and subjective relational distance) [82] rather than objective physical proximity [63]. On the other hand, our finding aligns with research suggesting that both in-person (e.g., patients’ suffering) and remote demanding interpersonal contacts (e.g., pressure to be available all the time) are associated with impaired productivity and wellbeing [1,40].

Overall, our findings contribute to fill the literature gap in (a) the conceptualization of employee psychological detachment *when at work* by addressing similarities and distinctiveness between workplace disconnectedness and work-related cynicism, (b) the investigation of productivity and wellbeing consequences of employee workplace disconnectedness, as well as its differential detrimental effects in comparison to a different form of employe detachment (i.e., work cynicism), and (c) the study of the role of different working arrangements (i.e., in-person vs. flexible) as a moderating factor of the effects of employee detachment on their productivity and wellbeing.

### 5.2. Practical Implications

The findings of this study have several practical implications. Our results suggest that increased employee workplace disconnectedness and work cynicism are both associated with impaired productivity and more distress, as well as higher difficulties in managing the work–family interface. Noteworthily, the toxic effects of workplace disconnectedness are specular and in line with previous research [32], suggesting that, when employees are filled with a high sense of belonging to their organization, this is associated with a 56% increase in job performance, as well as a 167% increase in their willingness to recommend the company to others (i.e., employer promoter score). Similarly, scholar and practitioners have warned against the post-pandemic unwanted effects for business and workforce health of employees’ cynical attitude in response to overwhelming emotional demands [66,85]. As such, organizations are advised to safeguard a positive and inclusive social ambience that may prevent employees’ defensive and detached reactions toward their community and their work. Interventions aimed at preventing workplace disconnectedness and work cynicism may include initiatives at the organizational and individual levels.

At the organizational level, training programs aimed at preventing disconnectedness and boosting belongingness may instead target supervisors and assist them in developing skills that are attentive to employees’ sense of belonging while simultaneously avoiding practices that impede inclusion. For example, inclusive leadership skills may be advanced by raising supervisors’ knowledge on (a) the complexities of cultivating employees’ sense of belonging by balancing their need to be accepted by others and prioritizing relationship building, as well as valuing the individual’s unique contribution, (b) employees’ need to be valued through meaningful actions that resonate with them in order to successfully promote belongingness, (c) the relevance of providing employees feedback to aid them in becoming mindful of their identity within the organization and in their relationships with others, thus improving employee–organization fit and fostering belongingness, and (d) the dynamics of belongingness development in newcomers who begin their relationship with the employer as outsiders who likely experience feelings of exclusion. Nevertheless, such dynamics of acceptance and inclusion may be facilitated by the leader in order to avoid newcomers’ insecurity that, in turn, may lead to a sense of not belonging. Relatedly, organizational level interventions aimed at preventing employee burnout and cynical attitude toward others and work may target supervisors and their leadership styles. Indeed, supervisor support and leadership are thought to be essential work resources capable of reducing burnout in employees, and several studies have shown that authentic, transformational, and servant leadership styles have positive effects on employees’ psychological resources and are related to lower burnout levels [86]. As such, training programs may aim at developing these leadership styles in order to prevent the progressive chronicization of employees’ cynical behaviors. Moreover, leaders’ performance and leadership behaviors should also be regularly evaluated so that potentially adverse aspects may be identified in a timely manner to avoid triggering employee burnout [39]. Additionally, given that role conflicts and ambiguities are potential triggers of burnout, organizations should develop welcoming processes for new workers, wherein the mission of the job position and tasks are explained with clarity, and new employees should be progressively introduced to the most stressful elements of the job, always with the support of the supervisor or colleagues [87].

At the individual level, our findings may assist in defining project intervention by showing that belonging to something and the social ties that accompany a sense of belonging may help individuals feel they are not alone, representing a resource in effectively coping with difficulties and a protective factor to decrease the physical and mental effects of difficulties [47,52]. Relatedly, organizations may counteract the recent increase in employees’ feelings of isolation [3,88] and develop their inclusion in the workplace by programming interventions aimed at boosting employees’ sense of organizational connectedness through the mastery of strategies such as [47] (a) creating social occasions with coworkers, (b) being mindful of others by asking questions, making small talk, self-disclosing, and listening to coworkers, (c) practicing acceptance by acknowledging that coworkers are different from oneself and focusing on similarities that promote bonding, and (d) appreciating coworkers’ efforts to seek contact that helps in building the sense of connectedness. Relatedly, employee level interventions aimed at preventing the unfold of cynicism may include coaching and guidance as nondirective methods that encourage employees to regain control of their own emotional state and wellbeing. Specifically, the coach does not prescribe any treatment but instead guides employees to come up with coping strategies on their own [89]. Moreover, employees could be assisted in coping with the challenges of daily life and provided with peer and team support as an important resource. Support groups are defined as group of formal or informal coworkers that meet regularly (e.g., 2 h every 2 weeks) to give each other emotional support, exchange information, and/or solve work problems. The primary goal of support groups is to help individuals to reduce feelings of loneliness and emotional exhaustion, as well as provide occasions to exchange knowledge and develop effective ways of problem solving. The benefits of this type of intervention on burnout have been repeatedly demonstrated [90]. Interestingly, we note that this type of intervention targets employees’ loneliness that is coincidentally considered a factor that undermines their sense of belongingness and is potentially conducive to workplace disconnectedness, thus qualifying as an important tool in order to prevent employee detachment in terms of both disconnectedness and cynicism. Support groups encompass a wide range of activities, including the creation of formal support groups and the celebration of professional achievements. Organizations aiming at fostering a sense of community should incorporate activities into work processes such as dedicating time to share knowledge and ideas on how to deal with day-to-day challenges.

### 5.3. Strengths, Limitations, and Future Directions

While the current findings are promising and suggest that employees’ detachment in the form of both sense of disconnectedness from one’s organization and distance from one’s work may impair their wellbeing and productivity, as well as the work–family interface, they also warrant further investigation. Our study is an important first step in demonstrating that employee detachment may take different, but equally toxic, forms of psychological distancing from overwhelming experiences at work. Yet, future studies may further expand this framework. Given the social component inherently embedded in both workplace disconnectedness and cynicism, future research for advancing the literature on the link between employee detachment and its outcomes points at incorporating the study of how contextual factors influence psychosocial organizational processes in one’s workplace and their influence in shaping the individual experience of productivity and wellbeing. Indeed, the belongingness literature has highlighted that an organization’s culture impacts employees’ sense of belonging and related job happiness [91]. Similarly, the exercise of a role within an organization is embedded in specific norms of behavior that may contribute to posing pressure on employees and contribute to enhancing the level of stress in their job [92]. Moreover, culture is suggested to moderate numerous aspects of the interface between work and family domains, such as social regulations and policies in place to support families, and cultural gender-related norms related to children caregiving obligations, costs, etc. [93]. In light of this, future studies should consider the moderating role of organizational culture (e.g., culture of diversity and inclusion) on the nomological network tested in our study within a full multilevel modeling approach that examines employees nested within organizations and accounts for possible organizational differences. An additional venue for advancing our understanding of the social mechanisms underlying the emergence of employee feelings of exclusion (i.e., disconnectedness) and indifference (i.e., cynicism), as well as related health outcomes, may include the study of social identity processes that contribute to build workplace belongingness, as well as employee wellbeing, such as group–member prototypicality [26,94]. An additional strength of the current study is the multilevel data drawn from numerous organizational samples representing a wide variety of industries. Indeed, the recent literature [95] on the association of workplace stress with different industry sectors suggests that healthcare workers, social workers, and teachers experience higher levels of stress than workers in other professions; technology and finance industries are also particularly stressful due to factors such as high workloads, long working hours, and job insecurity. Noteworthily, the industry sectors with a larger percentage in our sample were healthcare (12*%*), education (19.8%), communication and technology (10.6%), and services and finance (5.3*%*), thus contributing to a representation of the most at-risk occupational settings in our research. Nonetheless, our data stemmed from a convenience sample; thus, our findings might be affected by self-selection bias. As such, future studies should attempt to include additional types of occupations, as well as organizational samples from diverse national contexts, in order to increase the generalizability of the present findings.

An arguable limitation is that this study relies on cross-sectional and self-report data. Indeed, individuals are the best informants when assessing constructs such as perceived wellbeing and stressors. Yet, future studies should use multisource measures of employee outcomes, such as health records whether available and supervisor’s ratings of performance and off-task cognitive performance (i.e., cognitive failures). This would reduce the likelihood of common method bias occurring in self-report data [96]. Moreover, while the use of cross-sectional data prevents causal conclusions, future longitudinal studies introducing temporal distance between predictors (i.e., workplace disconnectedness and cynicism) and outcomes could provide additional support for the causal links proposed in our model and theoretically driven. Moreover, additional longitudinal studies may aim at examining within-person processes (i.e., employee detachment) using latent growth curve models in order to examine via a parallel processes model whether concomitant increases or decreases in employees’ sense of disconnectedness (i.e., disconnectedness as a transient and mutable employee feeling) [97] and distant attitude toward work (i.e., cynicism) are associated with similar trends in workers’ productivity, wellbeing, and work–family interface across time.

Lastly, despite evidence suggesting that a lack of sense of belonging to a valued group builds upon the network of non-inclusive and unsupportive relationships provided by work [98] and undermines employee happiness [99], research on the psychosocial emotional processes underpinning the development of employees’ feelings of disconnectedness from their community is still scarce. Hence, future studies may examine how the spreading of emotional contagion rooted into environmental cues exchanged during interactions at work [100] contributes to the escalation of negative emotions (e.g., frustration and anger) that boost employee detachment and compromise the “we” feeling leading to employee wellbeing and engaged performance.

## 6. Conclusions

Employee detachment in the post-pandemic era is an increasing concern, also due to flexible work arrangements and related stay away from the workplace. The present study focuses on workplace disconnectedness (i.e., the employee feeling of lack of belongingness to their organization and inclusion in a valued community of coworkers) and work cynicism (i.e., a negative and excessively detached reaction in response to relational overload at work), examining across groups of in-person and flexible workers from 204 different organizations how employees’ workplace disconnectedness differs from their cynicism, and how disconnectedness and cynicism differentially exert their detrimental effects on employee performance, wellbeing, and work–family interface. Our results show a similar pattern of results for flexible and in-person workers. Specifically, compared to disconnectedness, cynicism exerted higher negative effects on mental health and higher positive effects on cognitive failures and family-to-work conflict. Conversely, compared to cynicism, disconnectedness exerted higher negative effects on performance and work-to-family conflict. Overall, our findings inform scholars and practitioners on the toxic effects of different forms of employee detachment and provide practical suggestions on how to attenuate their unwanted effects on employee productivity and wellbeing.

## Figures and Tables

**Table 1:** Descriptive statistics, correlations, and reliabilities.

Variable	Mean	SD	1	2	3	4	5	6	7
1. Workplace disconnectedness	1.72	0.76	0.91/0.91						
2. Cynicism	2.06	10.37	−0.55 ***	0.86/0.93					
3. In-role performance	4.51	0.69	−0.24 ***	−0.21 ***	0.84/0.88				
4. Cognitive failures	2.13	0.56	0.34 ***	0.39 ***	−0.28 ***	0.81/0.81			
5. Mental health	4.18	0.93	−0.43 ***	−0.49 ***	0.21 ***	−0.38 ***	0.76/0.78		
6. WFC	2.32	0.78	0.30 ***	0.28 ***	−0.09 ***	0.23 ***	−0.31 ***	0.61/0.63	
7. FWC	1.98	0.77	0.35 ***	0.38 ***	−0.25 ***	0.38 ***	−0.33 ***	0.50 ***	0.70/0.70

Note. Reliability estimates (maximal reliability and composite reliability, respectively) are on the diagonal. *** *p* < 0.001.

**Table 2:** Results of tests for measurement and structural invariance across in-person and flexible workers.

	Model Fit						Model Comparison		
Models(M)	SBχ^2^	*df*	RMSEA (90% CI)	CFI	TLI	SRMR	ΔM	∆SBχ^2^_(∆df)_	ΔCFI
Measurement models									
Model _in-person_	585.206	168	0.058 (0.053–0.063)	0.932	0.915	0.046	−		−
Model _flexible_	323.936	168	0.061 (0.051–0.071)	0.915	0.894	0.061	−		−
M1: Configural	913.868	336	0.059 (0.054–0.063)	0.928	0.910	0.050	−		−
M2: Metric	926.215	350	0.057 (0.053–0.062)	0.928	0.914	0.053	M1-M2	180.347_(16)ns_	0
Structural models									
S4: Unconstrained structural effects across groups with metric invariance	954.667	361	0.057 (0.053–0.062)	0.926	0.914	0.058	−		−
S5: Constrained structural effects across groups with metric invariance	926.217	350	0.057 (0.053–0.062)	0.928	0.914	0.053	S4-S5	280.487_(11)ns_	−0.002

Note. At each step in the sequence of invariance tests, all earlier constraints remained in place. *df* = degrees of freedom; RMSEA = root-mean-square error of approximation; CFI = comparative fit index; TLI = Tucker–Lewis index; SRMR = standardized root-mean-squared residual.

**Table 3:** Summary of results of hypothesis testing.

Hypothesis	Link	Effect (β)	Results
H1a	Disconnectedness → performance	−0.11 ***	Supported
H1b	Disconnectedness → cognitive failures	0.19 ***	Supported
H2a	Cynicism → performance	−0.21 ***	Supported
H2b	Cynicism → cognitive failures	0.33 ***	Supported
H3a	Disconnectedness → mental health	−0.24 ***	Supported
H3b	Cynicism → mental health	−0.43 ***	Supported
H4a	Disconnectedness → WFC	0.27 ***	Supported
H4b	Disconnectedness → FWC	0.24 ***	Supported
H5a	Cynicism → WFC	0.22 ***	Supported
H5b	Cynicism → FWC	0.33 ***	Supported

Note. *** *p* < 0.001.

## Data Availability

The data of the present study are unavailable as participants did not provide their permission to share.
